# Deubiquitinase USP39 and E3 ligase TRIM26 balance the level of ZEB1 ubiquitination and thereby determine the progression of hepatocellular carcinoma

**DOI:** 10.1038/s41418-021-00754-7

**Published:** 2021-03-01

**Authors:** Xiaomei Li, Jiahui Yuan, Conghua Song, Yongbin Lei, Jiajia Xu, Gongye Zhang, Weiwei Wang, Gang Song

**Affiliations:** Cancer Research Center, School of Medicine, Xiamen University, Xiamen, China

**Keywords:** Gene regulation, Prognostic markers

## Abstract

Emerging evidence suggests that USP39 plays an important role in the development of hepatocellular carcinoma (HCC). However, the molecular mechanism by which USP39 promotes HCC progression has not been well defined, especially regarding its putative ubiquitination function. Zinc-finger E-box-binding homeobox 1 (ZEB1) is a crucial inducer of epithelial-to-mesenchymal transition (EMT) to promote tumor proliferation and metastasis, but the regulatory mechanism of ZEB1 stability in HCC remains enigmatic. Here, we reveal that USP39 is highly expressed in human HCC tissues and correlated with poor prognosis. Moreover, USP39 depletion inhibits HCC cell proliferation and metastasis by promoting ZEB1 degradation. Intriguingly, deubiquitinase USP39 has a direct interaction with the E3 ligase TRIM26 identified by co-immunoprecipitation assays and immunofluorescence staining assays. We further demonstrate that TRIM26 is lowly expressed in human HCC tissues and inhibits HCC cell proliferation and migration. TRIM26 promotes the degradation of ZEB1 protein by ubiquitination in HCC. Deubiquitinase USP39 and E3 ligase TRIM26 function in an antagonistic pattern, but not a competitive pattern, and play key roles in controlling ZEB1 stability to determine the HCC progression. In summary, our data reveal a previously unknown mechanism that USP39 and TRIM26 balance the level of ZEB1 ubiquitination and thereby determine HCC cell proliferation and migration. This novel mechanism may provide new approaches to target treatment for inhibiting HCC development by restoring TRIM26 or suppressing USP39 expression in HCC cases with high ZEB1 protein levels.

## Introduction

Hepatocellular carcinoma (HCC) is a prevalent malignant tumor with high morbidity and mortality, respectively ranked as fifth and second among all cancers worldwide [1]. More precisely, 841,080 people from 18.1 million cancer cases and 781,631 deaths of 9.6 million cancer deaths were due to HCC in 2018 only [1]. Postoperative recurrence and metastasis are the major cause and obstacles to improve the prognosis of HCC patients, with a low overall 5-year survival rate [2]. The progression of HCC is associated with aberrant epithelial-to-mesenchymal transition (EMT), which triggers cellular mobility and subsequent dissemination of tumor cells [3]. The EMT program requires tight control at various levels, including posttranslational regulation of key proteins [4]. Coordination between ubiquitination and deubiquitination modifications of the ubiquitin–proteasome system (UPS) is an important posttranslational regulatory mechanism, responsible for the degradation and turnover of EMT-related proteins [5, 6].

Zinc-finger E-box-binding homeobox 1 (ZEB1) has been characterized as a crucial driver of tumor invasion, distant metastasis, drug resistance, and radioresistance by inducing the EMT in tumor epithelial cells [7–9]. Accumulating evidence indicates that ZEB1-mediated EMT plays a critical role in the development of HCC. It has been previously proposed that the dysregulation of the ubiquitination process is highly relevant to the aberrant degradation of ZEB1 [10] and EMT in carcinomas [11]. Although some existing clues indicate the role of UPS in mediating ZEB1 stability, studies of ZEB1 ubiquitination are still in the primary stage. Furthermore, there are few reports about the deubiquitination of ZEB1. Hence, the molecular mechanism of ZEB1 stability in tumor cells, which contribute to the progression of HCC, is not fully understood.

Ubiquitin-specific peptidase 39 (USP39), a member of the deubiquitylation family, contains both a central zinc-finger ubiquitin-binding domain and an ubiquitin C-terminal hydrolase domain [12]. An increasing amount of evidence has demonstrated that the aberrant expression of USP39 promotes tumorigenesis and is associated with HCC progression [13, 14]. Our previous studies have shown that overexpressed USP39 can promote the proliferation and migration of human ovarian cancer by targeting EMT [15]. Currently, USP39 is well known as an important modulator of RNA splicing [16, 17]. Intriguingly, USP39 is considered to be devoid of deubiquitinating activity due to the absence of three important active-site residues (i.e., cysteine, histidine, and aspartic acids) [18]. Nevertheless, it has been demonstrated that USP39 can regulate the stability of DNA damage-related protein CHK2 by deubiquitination, to further regulate cellular processes in the context of lung cancer [19]. Although the aberrant USP39 expression has been implicated in EMT during tumorigenesis, its molecular mechanisms in HCC remain unknown. Since ubiquitination and stability play important roles in ZEB1 regulation, hypothetically, USP39 with the function of deubiquitination could promote HCC progression through regulating the level of ZEB1 ubiquitination.

The tripartite motif (TRIM) family proteins possess one or two B-boxes along with its domain structure, and a more diverse domain at the C terminus [20, 21], which are considered key regulators of protein degradation through ubiquitylation. Dysregulation of TRIM family proteins, particularly TRIM26, has been also implicated in several cancers [22–24]. Current studies have indicated that TRIM26 is downregulated in several cancers [25, 26]. These studies have suggested that TRIM26 may act as a novel tumor suppressor of HCC since its downregulation contributes to a worse prognosis [24]. Moreover, overexpression of TRIM26 has been found to significantly inhibit cell proliferation, migration/invasion, and EMT phenotype in HCC [25]. TRIM26 has an E3 ubiquitin ligase activity, which is involved in the degradation of numerous target proteins. Nakagawa et al. reported that the E3 ubiquitin ligase TRIM26 ubiquitylated the TAF7 subunit of transcription factor IID in a TGF-β induced mechanism leading to mouse mammary epithelial cells proliferative arrest [27]. TRIM26 could also control the level of NEIL1 involved in the DNA damage response through direct ubiquitylation [28]. Nevertheless, the function of TRIM26 has not been well established in the progression of HCC. It is interesting to speculate that whether E3 ligase TRIM26 with ubiquitin function has a common foothold with USP39, that is, TRIM26 affects the stability of ZEB1 through ubiquitination, while USP39 promotes the abnormality of ZEB1 level through deubiquitination, which orchestrates the occurrence of EMT and determines the progression of HCC.

This study uncovers the elaborate ubiquitination and deubiquitination modifications of ZEB1 in HCC. Our data demonstrate that deubiquitinase USP39 and E3 ligase TRIM26 balance the level of ZEB1 ubiquitination and thereby determine the progression of HCC. In detail, USP39 inhibits ZEB1 degradation through its deubiquitination function to promote HCC progression and TRIM26 degrades ZEB1 via ubiquitination to exert tumor-suppressive functions. In contrast to the E3 ligase TRIM26, USP39 is highly expressed in clinical HCC samples, indicating a positive correlation with poor disease prognosis. In addition, a direct interaction between USP39 and TRIM26 was identified by co-immunoprecipitation and immunofluorescence staining assays, respectively. Collectively, our findings suggest that the balanced deubiquitination and ubiquitination of ZEB1 by USP39 and TRIM26 represent a novel mechanism for the progression of HCC, and this discovery provides a promising strategy for targeting USP39 or TRIM26 in the treatment of HCC cases with aberrant ZEB1 expression levels.

## Materials and methods

### Human HCC samples and tissue microarray

Human HCC samples, including HCC tissue and adjacent hepatic tissues, were obtained from 25 patients who underwent hepatectomy for primary HCC at the Affiliated Hospital of Putian University (Fujian, China) during January 2017 and January 2019. Tissue microarray of HCC tissue and adjacent hepatic tissues was purchased from Shanghai Outdo Biotech Co., Ltd, China.

### Cell lines and culture

The human HCC cell lines (SK-hep-1 and HepG2) were obtained from the Shanghai Cell Bank of the Chinese Academy of Sciences, China. HCC cells were cultured in modified Eagle’s medium (MEM) supplemented with 10% fetal bovine serum (FBS) and 1% penicillin/streptomycin. All cells were grown at 37 °C in a humidified incubator with 5% CO_2_.

### In vivo experiments

The nude mouse xenograft model: SK-hep-1 cells (4 × 10^6^) stably transduced with shUSP39 and shTRIM26 were injected subcutaneously into the flanks of 6–8-week-old male BALB/c nude mice, respectively. Tumor length (a) and width (b) were measured with a vernier caliper every 3 days, and then tumor volume was calculated using the formula: 1/2ab^2^. The mice were sacrificed on the 30th day after injection, and tumors were isolated and weighed.

Lung metastasis in the nude mouse model: USP39 and TRIM26 knockdown SK-hep-1 cells (1 × 10^6^) were respectively injected into the tail vein of nude mice. Tumor growth was monitored by whole-body bioluminescent imaging (photons/s/cm^2^/sr) with Bruker In Vivo FX PRO Imaging System (Bruker, Germany). Mice were anesthetized using isoflurane on the 40th day after injection. Then their lungs were removed and the numbers of surface adenomas were counted. Tumor tissues were stored at −80 °C and fixed with 4% paraformaldehyde for immunohistochemistry (IHC).

### Statistical analysis

Each experiment was repeated independently at least three times. Continuous variables were presented as mean and standard deviation (SD). Unpaired two-tailed student’s *t* test or one-way analysis of variance was employed for comparison of continuous variables. Pearson’s or Spearman’s correlation coefficient was used for evaluating the relationship between the two groups. All Analyses were performed using GraphPad Prism 8 software (GraphPad Software, USA). A *P* value of <0.05 was considered statistically significant. Asterisks (*, **, and ***) stand for *P* < 0.05, *P* < 0.01, and *P* < 0.001, respectively.

### Supplementary information

Detailed protocols and procedures are provided in the Supplementary Materials and Methods section.

### Human HCC samples and tissue microarray

Human HCC samples, including HCC tissue and adjacent hepatic tissues, were obtained from 25 patients who underwent hepatectomy for primary HCC at the Affiliated Hospital of Putian University (Fujian, China) during January 2017 and January 2019. These patients had not received any radiotherapy or chemotherapy treatment prior to hepatectomy. Tumor staging was defined according to the 8th Edition of the American Joint Committee on Cancer (AJCC8) Staging Manual [29]. Tissue microarray of HCC tissue and adjacent hepatic tissues was purchased from Shanghai Outdo Biotech Co., Ltd, China.

### Cell lines and culture

The human HCC cell lines (SK-hep-1 and HepG2) were obtained from the Shanghai Cell Bank of Chinese Academy of Sciences, China. HCC cells were cultured in MEM supplemented with 10% FBS. HEK 293T was cultured in Dulbecco’s modified Eagle’s medium containing 10% FBS and 1% penicillin/streptomycin. All cells were grown at 37 °C in a humidified incubator with 5% CO_2_. In separate experiments, cells were cultured in the presence of 0.2 mg/ml cycloheximide (CHX, an inhibitor of protein synthesis) or Z-Leu-Leu-Leu-al (MG-132, an inhibitor of the ubiquitin–proteasome pathway) [30, 31].

### Plasmid construction and gene transfection

Containing the full-length *trim26* fragment of pcmv-N-Myc plasmid (Myc-TRIM26) was constructed and sequenced. Flag-USP39 plasmid and the different domains of USP39 plasmids, as USP39-RS (1-104aa), USP39-ZNF (105-199aa) and USP39-USP (200-555aa), have been constructed in the pcmv14-N-Flag plasmid. Knockdown of USP39 or TRIM26 using shRNAs were generated from Genechem, China. Double-strand oligonucleotides corresponding to the RNA sequences were cloned into the pGV248-RNAi plasmid (Oligoengine, USA). Target sequences for human TRIM26 mRNA were as follows: shRNA#1 GATGGATATGACGACTGGGAA and shRNA#2 GCTGCTGAGAGACTTGGAATA. Target sequences for human USP39 mRNA were as follows: shRNA#1 TTCCAGACAACTATGAGAT and shRNA#2 TTTGGAAGAGGCGAGATAA. Then these plasmids were transfected respectively into the HCC cells using Lipotransfectamine 3000 (Thermo Fisher Scientific). Recombinant lentiviruses were produced by HEK 293T cells following the co-transfection of shRNAs with the packaging plasmids. After that, the supernatants containing lentiviruses were harvested and added onto cells in culture supplemented with 8 μg/ml polybrene.

### PCR analysis

Total RNA was extracted using the High Pure RNA Isolation Kit (Roche, Germany) and reverse transcription-PCR was performed with the PrimeScript RT Reagent Kit Perfect Real-Time (Takara, China). Quantitative RT-PCR was conducted to detect the levels of mRNA using the Takara’s SYBR Premix Ex Taq^TM^ II (Takara, China). Relative fold-change of target genes expression was normalized to the abundance of GAPDH and estimated by the 2^−ΔΔCq^ method. The specific primer sequences were listed in Supplementary Table S1. Thermal conditions for PCR reactions were as follows: 95 °C initial denaturation step for 5 min, 40 cycles of denaturation at 95 °C for 10 s, primer annealing at 58 °C for 20 s, and extension at 72 °C for 20 s. All reactions were performed in triplicate.

### Western blot analysis

Cell lysates were prepared in RIPA protein extraction buffer (Beyotime Institute of Biotechnology, China) with protease inhibitors and phosphatase inhibitor cocktail (Roche, Germany). Proteins in lysates were separated by SDS-polyacrylamide gel electrophoresis and then transferred onto a polyvinylidene fluoride membrane (EMD Millipore Billerica, USA). Membrane was blocked with 5% nonfat milk and incubated with specific primary antibodies overnight at 4 °C. It was then incubated with horseradish peroxidase-conjugated secondary antibody for 1 h at 37 °C. Finally, the protein signals were visualized and quantified using Electrochemiluminescence (Thermo Fisher Scientific, USA) and ChemiDoc^TM^ MP Imaging System (Bio-Rad Laboratories, USA). Primary antibodies and corresponding manufacturers were showed as follows: TRIM26 (ab89290, 1:1000) and USP39 (ab131244, 1:1000) (Abcam, USA); N-cadherin (Rabbit mAb#13116, 1:1000), Ki-67 (mAb #9449) and snail (Rabbit mAb #3879, 1:1000) (Cell Signaling, USA); ZEB1 (22018-1-AP) (Proteintech, USA); mouse monoclonal antibodies against Flag and HA (OriGene, USA); and β-actin (1:40,000) (Sigma-Aldrich, USA).

### Cell proliferation and colony formation assay

HCC cell viability was detected using a cell proliferation assay kit (Beyotime, China). Cells were seeded into 96-well plates at a density of 5000 cells/well. At indicated time points, 20 μL of MTT reagent (Promega, USA) was added to each well and then incubated for 4 h at 37 °C. The cell proliferation curves were determined by measuring optical density (OD) at a wavelength of 570 nm.

In colony formation assay, about 500 cells were cultured in 6-well plates and maintained in media containing 10% FBS at 37 °C for 12 days. Then the colonies were fixed with 4% polyoxymethylene and stained with crystal violet (Sigma-Aldrich, USA). The colony counts were normalized to the control and expressed as a percentage.

### Transwell assay and wound-healing assay

The transfected cells were harvested at 24 h, and then 5 × 10^4^ cells suspended in serum-free medium were seeded in the upper chambers of 24-well Transwell. And the upper chambers for invasion assays were coated with Matrigel (Sigma-Aldrich; Merck KGaA). Then medium containing 20% FBS was added into the lower chambers. After incubation for 18 h at 37 °C, the cells remaining on the upper surface of the membrane were removed with a cotton swab. Migrated and invaded cells were fixed in methanol, stained in 0.1% crystal violet, and then quantified from microscope at ×4 magnification.

In the wound-healing assay, transfected HCC cells were seeded in 6-well plates and cultured overnight. The “scratch” wounds were created by scraping the cell layer with a sterile 200 μl pipette tip. Cells were cultured in media containing 2% FBS for another 24 h and imaged under a microscope.

### Immunoprecipitation

Immunoprecipitation assay was performed as described in Lv et al. [32]. Cells were lysed in NP-40 lysis buffer. The protein lysate (5%) was analyzed by western blot analysis (input). Then the remaining lysate was added to protein A/G agarose beads pre-coupled with antibody and incubated at 4 °C for 4 h. The beads were washed and then boiled in 2 × SDS loading buffer for western blotting analysis.

### In vivo ubiquitination assay

Cells were transfected with Flag/HA-Ubiquitin and indicated plasmids for in vivo ubiquitination assay, which was performed as previously described [33].

### GST pull-down assay

The sequences encoding TRIM26 were cloned into a pGEX-4T-1 vector plasmid (GST-TRIM26). GST-TRIM26 and GST-vector plasmids were transfected into *E. coli*. BL21 and purified as previously described [34]. Whole cell lysates (containing HA-Ub and Flag-ZEB1 proteins) were prepared in HEK 293T cells. Whole cell lysates were incubated with GST-TRIM26 fusion protein solution for 4 h at 4 °C, and the GST-vector was used as the control. The bound proteins were analyzed using the immunoblotting assay.

### Immunofluorescence and immunohistochemistry

Cells were fixed with 4% paraformaldehyde for 20 min and permeabilized with 0.1% Triton X-100 (Sigma-Aldrich, USA) for 5 min. Samples were blocked with 5% bovine serum albumin in PBS for 1 h, and then incubated with primary antibodies at 4 °C overnight, followed by incubation with fluorescence-labeled rabbit/mouse secondary antibody (Invitrogen, USA) for 1 h at room temperature. Nuclei were stained with DAPI (Invitrogen, USA) for 10 min and observed with a fluorescence microscope (Olympus, Japan).

Immunohistochemical staining was performed as previously described [35]. Paraffin sections (4 μm thick) were first incubated in an oven at 60 °C for 2 h, then deparaffinized with xylene and rehydrated through gradient ethanol immersion. Boiled for antigen retrieval with 0.01 mol/L citrate buffer solution (pH 6.0) for 30 min. Endogenous peroxidase blocking reagent (Cell Marque, Rocklin, CA) was added for 10 min to block non-specific antibody binding. Sections were also blocked in 1% fetal calf serum for 10 min, and then incubated with the primary antibody in humidified chamber overnight at 4 °C, and treated with a streptavidin-perosidase-conjugated second antibody (Fuzhou Maixin Biotech. Co., Ltd, China) for 30 min at room temperature. The immune complexes were visualized with a Vectastatin DAB kit (Vector Laboratories). Following staining, five high power fields in each slide at ×40 magnification were scored by two examiners. The proportion score was calculated as follows: 0 (no positive tumor cells), 1 (<25% positive tumor cells), 2 (25–50% positive tumor cells), 3 (50–75% positive tumor cells), and 4 (>75% positive tumor cells). Staining intensity was graded according to the following criteria: 0 (no staining), 1 (weak staining, light yellow), 2 (moderate staining, yellow-brown), 3 (strong staining, brown), and 4 (strong staining, dark brown). Staining index (SI) was calculated as the product of staining intensity score and the proportion of positive tumor cells. An SI score of ≥6 was used to define tumors with high expression, and ≤4 as tumors with low expression.

### Kaplan–Meier plotter analysis

Kaplan–Meier plotter (http://www.kmplot.com) was used to generate overall survival (OS) curves of USP39 and TRIM26 in HCC. Expression analysis was conducted using publically available RNA-seq data and follow-up information of 364 HCC patients from the project of the Cancer Genome Atlas (TCGA). Using the median risk score in TCGA data as the cutoff value, HCC patients were divided into high-risk and low-risk groups.

## Results

### USP39 is highly expressed in human HCC tissues and correlated with poor prognosis

**Fig. 1:**
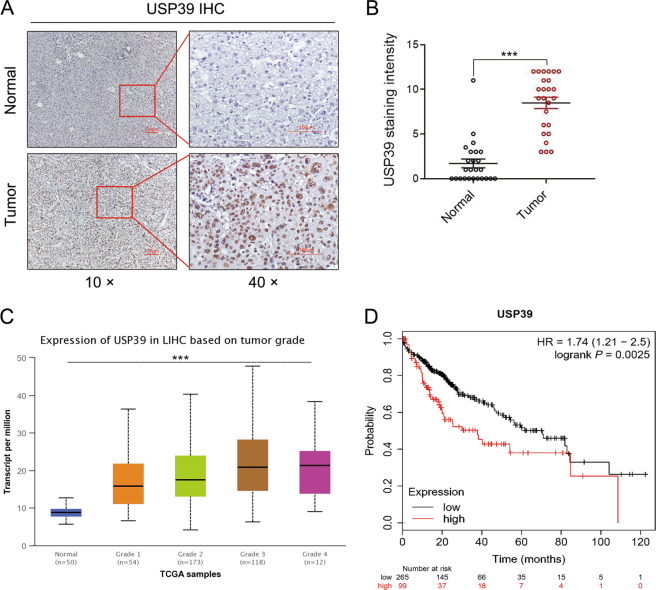
USP39 is highly expressed in human HCC tissues and correlated with poor prognosis. **A** Representative images (magnification, ×10 and ×40) of IHC staining for USP39 in HCC tissues and normal adjacent tissues from 25 patients. **B** Relative IHC staining for USP39 in HCC tissues and normal adjacent tissues (*n* = 25; ***P < 0.001). **C** Expression of USP39 is associated with tumor stage in HCC patients (***P < 0.001). **D** USP39 mRNA expression correlated with overall survival (OS) of HCC patients.

To assess whether USP39 plays an essential role in the development of HCC, IHC analysis was performed to detect the USP39 expression level in 25 human HCC samples. The analysis showed that USP39 protein displayed a higher expression level in most clinical HCC samples when compared to normal adjacent tissues from the 25 patient samples (Fig. 1A, B). USP39 positive staining was observed in the cell nucleus of HCC tissues. We also assessed the correlation between USP39 expression and 364 HCC patients’ clinicopathological parameters such as tumor grade and prognosis based on the TCGA database. As shown in Fig. 1C, USP39 expression was increased with advanced clinical grades of HCC. Moreover, Kaplan–Meier survival analysis revealed that HCC patients with high USP39 expression had a worse OS than those with low USP39 expression (Fig. 1D). The median OS time of HCC patients with high USP39 expression was ~37.8 months, which was markedly shorter than those with low USP39 expression (~70.5 months). These results indicated a potentially critical role of USP39 in the development of HCC and suggested that USP39 may be a valuable prediction factor for poor prognosis in HCC.

### USP39 promotes HCC proliferation and migration in vitro

**Fig. 2:**
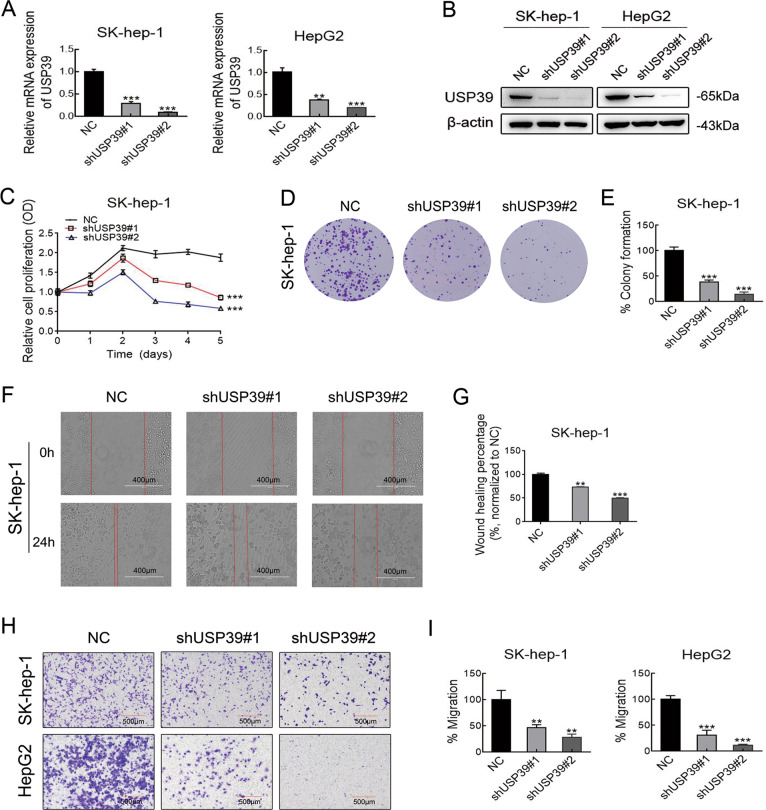
USP39 promotes HCC proliferation and migration in vitro. **A** The expression of USP39 mRNA was analyzed by RT-PCR in SK-hep-1 and HepG2 HCC cells infected with shRNAs. **B** Western blotting analysis of USP39 protein level in SK-hep-1 and HepG2 cells infected with shRNAs. **C**–**E** The effect of USP39 on HCC cells (SK-hep-1) proliferation was determined by MTT assays (**C**) at different time points and colony formation (**D**). The colony counts were normalized to the control and expressed as a percentage, and results are represented in the bar graph (**E**). **F**–**I** Representative images of HCC cell migration ability as shown by wound-healing assays (**F**–**G**) and migration assay (**H**–**I**). Student’s *t* test: *P < 0.05; **P < 0.01; ***P < 0.001. All the data are representative of at least three independent experiments and presented as the means ± SD.

To confirm the role of USP39 in HCC progression, we constructed HCC cell lines (SK-Hep-1 and HepG2) with stable USP39 downregulation using a lentiviral shRNA approach. The knockdown efficiency of USP39 was confirmed by RT-PCR and western blotting (Fig. 2A, B). MTT and colony formation assays were performed to assess the effect of USP39 knockdown on cell proliferation. The MTT results showed that silencing USP39 expression significantly inhibited the proliferative ability of HCC cells (Figs. 2C and S1). Consistently, colony formation assays demonstrated that USP39 knockdown dramatically restrained the ability of cells to form colonies (Fig. 2D, E). Wound-healing and transwell assays confirmed that USP39 knockdown remarkably suppressed the migration capacities of HCC cells (Fig. 2F–I). These results suggested that USP39 contributed to the protumorigenic effect of HCC as indicated by increased proliferation and migration.

### USP39 promotes EMT and inhibits ZEB1 ubiquitination in HCC

**Fig. 3:**
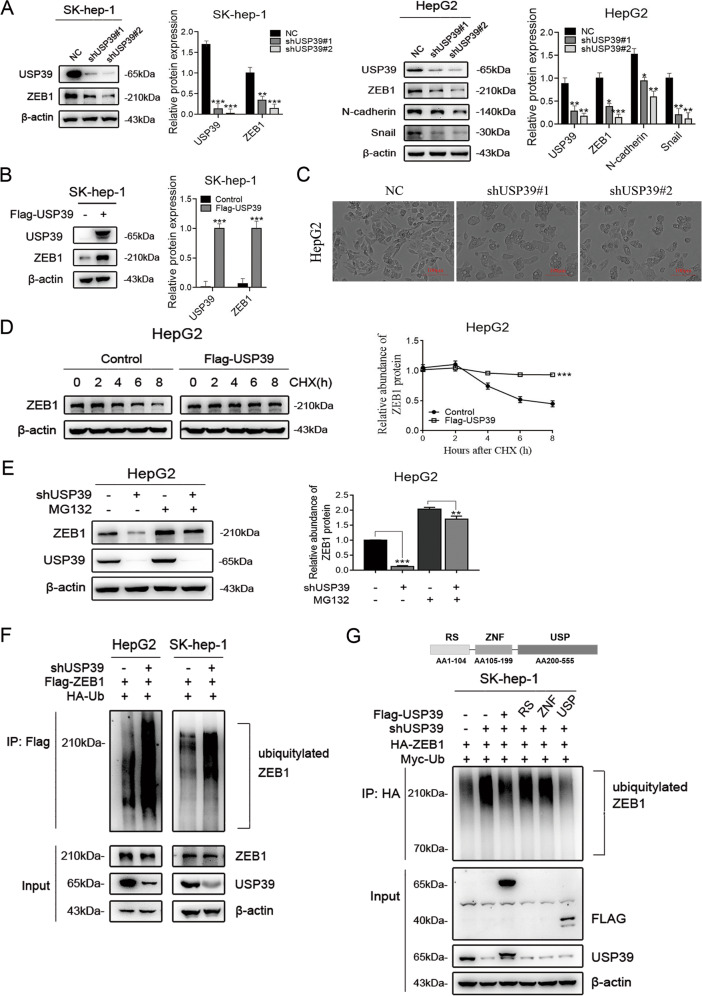
USP39 promotes EMT and inhibits ZEB1 ubiquitination in HCC. **A** The levels of USP39, ZEB1, N-cadherin and snail in HCC cells (SK-hep-1 and HepG2) with stable USP39 downregulation were analyzed by western blotting. **B** Effect of USP39 overexpression on the expression level of ZEB1 was confirmed by western blotting in SK-hep-1 cell. **C** Representative cellular morphology change of HepG2 cell after USP39 downregulation. **D** Quantitation of ZEB1 in HepG2 cells transfected with USP39 expressing plasmid was monitored by western blotting at the indicated times after cyclohexamide (CHX, 0.2 mg/ml) addition. Signal for ZEB1 was quantified densitometrically and relative aboundance of ZEB1 protein at the time of CHX addition (0 h) was set to 1. **E** The protein level of ZEB1 in HepG2 cell transfected with shUSP39 and treated with MG132 as indicated. **F** ZEB1 ubiquitination in USP39-knockdown HepG2 and SK-hep-1 cells co-transfected with Flag-ZEB1 and HA-Ub. **G** ZEB1 ubiquitination in USP39-knockdown HepG2 cells co-transfected with different domains of USP39 and the indicated plasmids. The transfected cells were treated with MG132 (20 μM for 4 h) prior to harvest. Student’s *t* test: *P < 0.05; **P < 0.01; ***P < 0.001. All the data are representative of at least three independent experiments and presented as the means ± SD.

Aberrant EMT induced by ZEB1 is involved in the proliferation and migration of HCC [36, 37]. To verify the effects of USP39 in this program, we further investigated whether USP39 affected the expression of ZEB1 and EMT-related markers (N-cadherin and Snail) in HCC cells (SK-hep-1 and HepG2). Our results revealed that the silencing of USP39 expression significantly suppressed ZEB1 protein expression in SK-hep-1 and HepG2 cells (Fig. 3A). Accordingly, the protein level of ZEB1 could be increased by USP39 overexpression in SK-hep-1 cell (Fig. 3B). These results suggested that ZEB1 protein expression positively correlated with USP39 protein expression. In agreement with the level of ZEB1 protein expression, N-cadherin and Snail expression were significantly decreased following USP39 knockdown in HepG2 cells (Fig. 3A). In addition, USP39 downregulation could inhibit the EMT phenotype in HCC cells (Fig. 3C). The control group of HepG2 cells exhibited a spindle-like mesenchymal phenotype, while the cells transduced with either shUSP39#1 or shUSP39#2 displayed cobblestone-like characteristics and cluster formation. This result strongly indicates that USP39 affect cell adhesion, which is an important feature of EMT. Collectively, these results revealed a functional significance of USP39 on the ZEB1 induced EMT progression in HCC at protein levels.

Interestingly, the ZEB1 mRNA level showed no significant difference in HepG2 cells transduced with shUSP39 (Fig. S2), which indicated that USP39 may mediate ZEB1 expression via posttranslational regulation (i.e., protein deubiquitination). However, this result conflicts with the previous report that USP39 has no deubiquitination function [18, 19]. Hence, we further tested whether USP39 could act as a deubiquitinase to regulate ZEB1 degradation. To verify the inferred mechanism of USP39 on the regulation of ZEB1 deubiquitination, we treated cells with cycloheximide (CHX, an inhibitor of protein synthesis) and monitored ZEB1 expression by western blotting at different time points. The results indicated that USP39 overexpression largely increased the half-life of the ZEB1 protein in HepG2 cells (Fig. 3D). In addition, downregulation of ZEB1 was dramatically reversed in USP39 knockdown HCC cells (HepG2) treated with MG132 (an inhibitor of the ubiquitin–proteasome pathway) (Fig. 3E). Clearly, the downregulation of USP39 greatly promoted ZEB1 ubiquitination in HepG2 and SK-hep-1 cells (Fig. 3F). Moreover, in order to study the key catalytic residues of USP39 for functioning as an active deubiquitinase (DUB), USP39 knockdown SK-hep-1 cells transfected with different domains of USP39. As shown in Fig. 3G, the ZEB1 ubiquitination was significant decreased in the cell transfected with USP domains of USP39. Together, these results identified that USP39 has a direct deubiquitinating activity towards the regulation of ZEB1 protein.

### Deubiquitinase USP39 interacts with the E3 ligase TRIM26

**Fig. 4:**
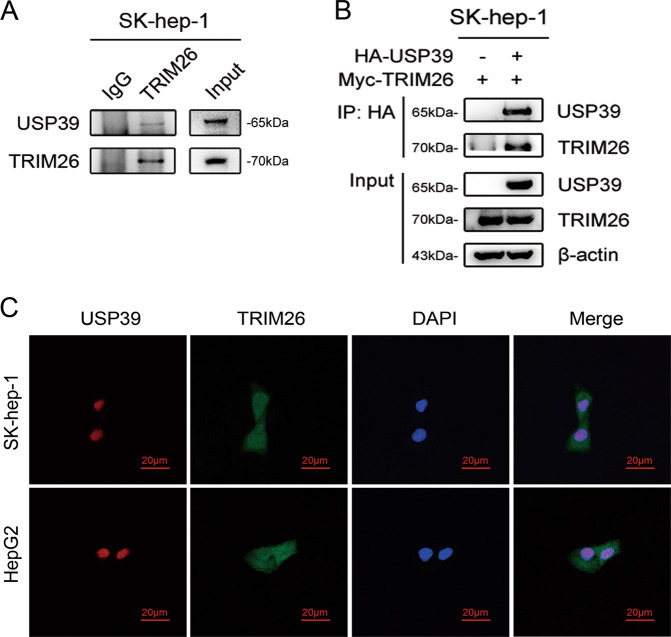
Deubiquitinase USP39 interacts with the E3 ligase TRIM26. **A** Co-immunoprecipitation assays showed an interaction between endogenous USP39 and TRIM26 in SK-hep-1 cell. Immunoglobulin (Ig) G was used for comparison as a negative control. Whole cell lysates for input were directly subjected to IB using antibodies. **B** Interaction of exogenous USP39 and TRIM26 in SK-hep-1 cell. HA-flag antibody was immunoprecipitated, and USP39 bound to TRIM26 was determined using immunoblotting (IB) with an anti-TRIM26 antibody. **C** Immunofluorescence staining assays (magnification, ×80) of USP39 and TRIM26 in cells (SK-hep-1 and HepG2) observed by confocal microscopy.

TRIM26 has an E3 ubiquitin ligase activity, which is involved in the degradation of numerous target proteins and may act as a novel tumor suppressor of HCC. It is interesting to speculate whether E3 ligase TRIM26 with ubiquitin function has an interaction with deubiquitinase USP39. Co-immunoprecipitation assays showed a direct interaction between endogenous USP39 and TRIM26 in SK-hep-1 cells (Fig. 4A). To further confirm the interaction between USP39 and TRIM26, co-transfection with HA-USP39 and Myc-TRIM26 expressing plasmids were conducted in SK-hep-1 cell. Results showed that ectopic expression of USP39 could interact with exogenous TRIM26 (Fig. 4B). Moreover, immunofluorescence staining assays revealed that USP39 and TRIM26 co-localized in the cell nucleus of hepatocyte (SK-hep-1 and HepG2), as observed by confocal microscopy (Fig. 4C). These observations suggested that deubiquitinase USP39 could interact with E3 ligase TRIM26 to affect the progression of HCC.

### TRIM26 is lowly expressed in human HCC tissues and inhibits HCC cells proliferation and migration

**Fig. 5:**
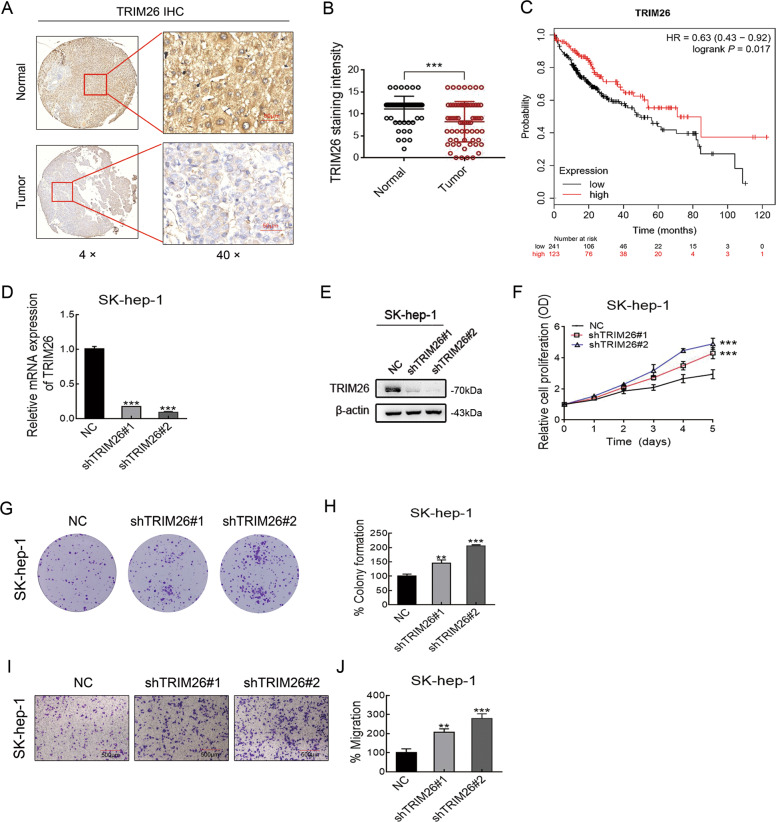
TRIM26 is lowly expressed in human HCC tissues and inhibits HCC cells proliferation and migration. **A** Representative images (magnification, ×40) of IHC staining for TRIM26 in HCC tissues and normal adjacent tissues. **B** Relative IHC staining for TRIM26 in HCC tissues and normal adjacent tissues. **C** TRIM26 mRNA expression correlated with overall survival (OS) of HCC patients. **D** The expression of TRIM26 mRNA was analyzed by RT-PCR in SK-hep-1 infected with shRNAs. **E** Western blotting analysis of TRIM26 protein level in SK-hep-1 cells infected with shRNAs. **F** MTT assay in SK-hep-1 cells transfected with shTRIM26. **G**, **H** Colony formation of SK-hep-1 HCC cells transfected with shTRIM26 (**G**). The colony counts were normalized to the control and expressed as a percentage, and results are represented in the bar graph (**H**). **I**, **J** Representative images (**I**) and Graphic representation (**J**) of the migration capacities in the TRIM26 knockdown SK-hep-1. Data are shown as the mean ± SD of three independent experiments. Student’s *t* test: *P < 0.05; **P < 0.01; ***P < 0.001.

To validate the role of TRIM26 in HCC, we also investigated the expression profiles of TRIM26 in clinical HCC samples (*n* = 75). Microarray expression profiling showed that the expression of TRIM26 was lower in most HCC tissues when compared with normal adjacent tissue (Fig. 5A, B). Immunofluorescence assays observed that TRIM26 positive staining was exhibited in the cell cytoplasm and nucleus. Furthermore, Kaplan–Meier survival analysis revealed that HCC patients with high TRIM26 expression had a better OS than those with low TRIM26 expression (Fig. 5C). These results indicated that TRIM26 can act as a valuable biomarker for identifying the prognosis of HCC patients.

Since TRIM26 participates in various biological processes, additional experiments were conducted to investigate the role of TRIM26 in HCC cell (SK-hep-1) transfected with TRIM26 shRNA. As shown in Fig. 5D, E, RT-PCR and western blotting confirmed that the gene knockdown mediated by TRIM26 shRNA was efficient in transfected cells. MTT assays demonstrated that the downregulation of TRIM26 significantly increased the proliferation capacity of SK-hep-1 (Fig. 5F). Furthermore, colony formation assays revealed that the TRIM26 knockdown was associated with a significantly increased clone number when compared with control SK-hep-1 cells (Fig. 5G, H). Thereafter, we investigated whether TRIM26 could affect HCC cell migration. Transwell assays revealed that the downregulation of TRIM26 expression could enhance cell migration capacities in SK-hep-1 (Fig. 5I, J). Taken together, these results suggest that TRIM26 can inhibit the progression of HCC by cell proliferation and migration.

### TRIM26 promotes the degradation of ZEB1 protein by ubiquitination in HCC

**Fig. 6:**
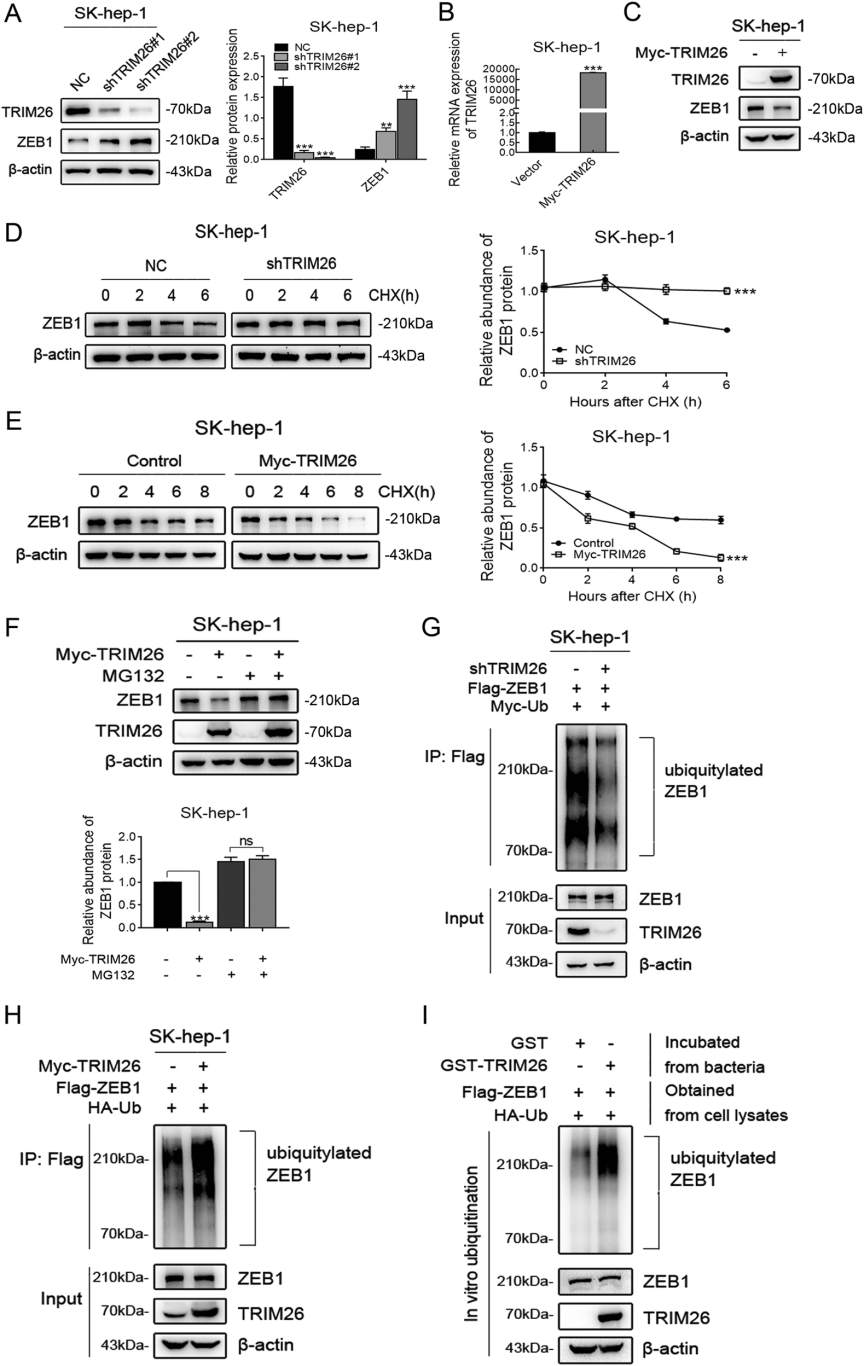
TRIM26 promotes the degradation of ZEB1 protein by ubiquitination in HCC. **A** Effect of TRIM26 knockdown on the expression of ZEB1 in SK-hep-1 cells as detected by western blot. **B** The mRNA expression of TRIM26 in TRIM26-transfected SK-hep-1 cell was determined by RT-PCR. **C** Effect of TRIM26 overexpression on the protein level of ZEB1 in SK-hep-1 cell as detected by western blot. **D** ZEB1 protein level in SK-hep-1 cells by downregulating TRIM26 at the indicated times after CHX (0.2 mg/ml) addition. **E** The amount of ZEB1 in SK-hep-1 cells transfected with TRIM26 expressing plasmid were monitored by western blotting at the indicated times after CHX (0.2 mg/ml) addition. **F** Levels of ZEB1 in TRIM26 over-regulated SK-hep-1 cells treated with MG132 (20 μM). **G** The ubiquitination of ZEB1 in TRIM26 knockdown SK-hep-1 cells co-transfected with expression plasmids encoding Flag-ZEB1 and HA-Ub. The transfected cells were treated with MG132 (20 μM for 4 h) prior to harvest. **H** The ubiquitination of ZEB1 by TRIM26 overexpression in SK-hep-1 cells. **I** ZEB1 ubiquitination by TRIM26 in vitro using purified proteins. Whole cell lysates (containing HA-Ub and Flag-ZEB1 proteins) was incubated with purified GST-TRIM26 or GST-Vector in vitro and blotted with TRIM26 or ZEB1 antibodies. Student’s *t* test: *P < 0.05; **P < 0.01; ***P < 0.001. All the data are representative of at least three independent experiments and presented as the means ± SD.

TRIM26 can inhibit the proliferation and migration of HCC, so it is necessary to investigate the effect of TRIM26 on the expression of ZEB1 protein. As shown in Fig. 6A, ZEB1 expression was significantly up-regulated after the TRIM26 knockdown in SK-hep-1. Moreover, the level of TRIM26 mRNA was significantly up-regulated following transfection of the TRIM26 overexpression vector in SK-hep-1 cell (Fig. 6B). Consistently, TRIM26 overexpression could lead to a decrease in ZEB1 expression in SK-hep-1 cell (Fig. 6C). Altogether, these results suggested that TRIM26 could regulate the stable expression of ZEB1 protein in HCC.

TRIM26 has an E3 ubiquitin ligase activity, which is involved in the degradation of numerous target proteins. Meanwhile, it is no significant difference in the ZEB1 mRNA levels from SK-hep-1 cells transduced with shTRIM26 (data not shown). Therefore, we speculated whether the regulatory effect of TRIM26 on ZEB1 is also through ubiquitylation. We treated cells with CHX and monitored ZEB1 expression by western blotting at different time points. The results indicated that the silencing of TRIM26 expression in SK-hep-1 significantly increased the half-life of ZEB1 protein (Fig. 6D), while ZEB1 stability largely decreased following TRIM26 overexpression in SK-hep-1 cells (Fig. 6E). Furthermore, TRIM26-induced protein reduction of ZEB1 was prominently reversed by the treatment with MG132 in SK-hep-1 cells (Fig. 6F). These results showed that TRIM26 destabilize ZEB1 protein through the proteasome pathway. Then, the TRIM26 knockdown dramatically suppressed the ubiquitination of ZEB1 in SK-hep-1 cell co-transfected with expression plasmids encoding Flag-ZEB1 and HA-Ub, in the presence of MG132 (Fig. 6G). And the ubiquitination of ZEB1 was remarkably enhanced by TRIM26 overexpression in SK-hep-1 cells (Fig. 6H). As shown in Fig. 6I, the GST pull-down assay demonstrated that TRIM26 could ubiquitylate ZEB1 in vitro. Together, these results confirmed our speculation that TRIM26 promotes the degradation of ZEB1 protein by ubiquitination in HCC.

### Deubiquitinase USP39 and E3 ligase TRIM26 balance the level of ZEB1 protein

**Fig. 7:**
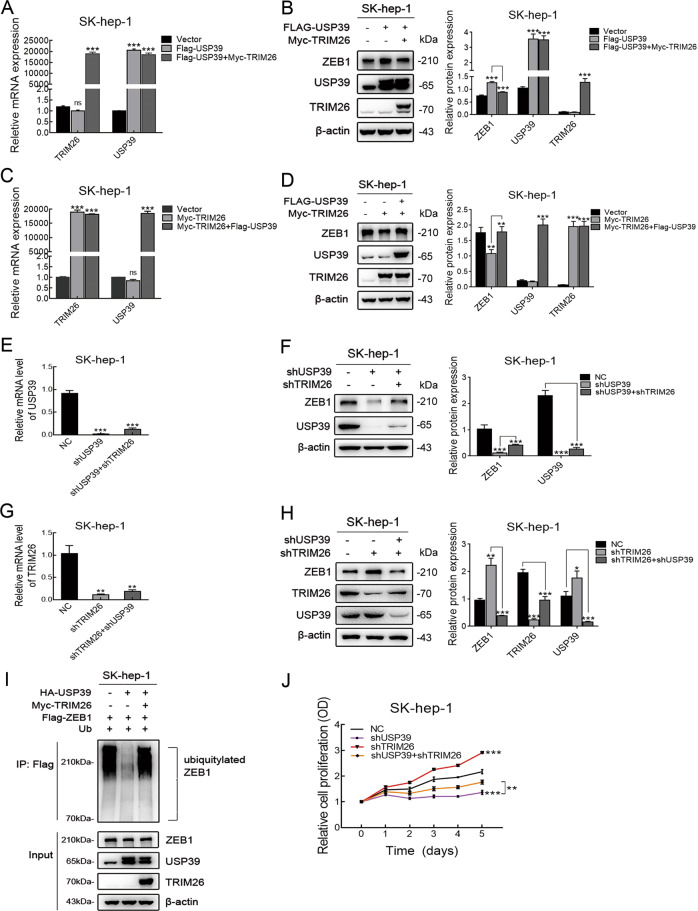
Deubiquitinase USP39 and E3 ligase TRIM26 balance the level of ZEB1 protein. **A**–**D** RT-PCR and western blotting indicated that the mRNA expression levels of USP39 and TRIM26 (**A**, **C**) and the protein level of ZEB1 (**B**, **D**) in SK-hep-1 cells co-translated with USP39 and TRIM26 expressing plasmids. (**E**, **G**) The mRNA levels of USP39 and TRIM26 were analyzed by RT-PCR in SK-hep-1 HCC cells. **F**, **H** Effect of USP39-knockdown and TRIM26 silencing on the protein level of ZEB1 in SK-hep-1 HCC cell determined by western blotting. **I** Effect of overexpression of USP39 and TRIM26 on the ZEB1 ubiquitination in SK-hep-1 cells. **J** Effect of USP39-knockdown and TRIM26 silencing on the cell proliferation of SK-hep-1 assessed by MTT assay. Student’s *t* test: *P < 0.05; **P < 0.01; ***P < 0.001. All the data are representative of at least three independent experiments and presented as the means ± SD.

As mentioned above, we found that USP39 and TRIM26 not only interact with each other but also have a common target (ZEB1 protein). Base on deubiquitinase USP39 and E3 ligase TRIM26 play an important role in modulating ZEB1 ubiquitination, we imagine that USP39 and TRIM26 could balance the ZEB1 expression to determine the progression of HCC. Firstly, to verify the regulatory relationship between USP39 and TRIM26 on ZEB1 expression, co-transfection with USP39 and TRIM26 expressing plasmids was conducted in SK-hep-1 cells. The overexpression efficiency of USP39 and TRIM26 was confirmed by RT-PCR and western blotting (Fig. 7A–D). As shown in Fig. 7B, the protein level of ZEB1 was increased after overexpression of USP39, and this effect could be abolished by overexpressing TRIM26. Accordingly, a decreased level of ZEB1 was detected in SK-hep-1 cell after TRIM26 overexpression, and this effect could be reversed by USP39 upregulation (Fig. 7D). This result confirmed that deubiquitinase USP39 and E3 ligase TRIM26 could balance the level of ZEB1 protein.

Considering that the ZEB1 protein is the common target of USP39 and TRIM26, we next sought to determine whether this equilibrium mechanism was associated with the progression of HCC. SK-hep-1 cells were transfected with shUSP39, shTRIM26, and shUSP39 + shTRIM26, respectively. Then, the efficiency of USP39 and TRIM26 deletion was confirmed by RT-PCR and western blotting (Fig. 7E–H). Consistently, the downregulation of TRIM26 reversed the ZEB1 protein level, which was significantly decreased in SK-hep-1 by shUSP39 transfection (Fig. 7F). Conversely, USP39 knockdown significantly reversed the ZEB1 protein level in SK-hep-1 after transfection with shTRIM26 (Fig. 7H). These results further supported that deubiquitinase USP39 and E3 ligase TRIM26 could balance the level of ZEB1 protein. Moreover, the ZEB1 ubiquitination was decreased after overexpression of USP39, and this effect could be reversed by TRIM26 overexpression in SK-hep-1 cells (Fig. 7I). Based on MTT results, cell proliferation was decreased by downregulating USP39 in SK-hep-1, and this effect could also be reversed by TRIM26 knockdown (Fig. 7J). Simultaneously, USP39 knockdown inhibited the proliferation of HCC cells transfected with shTRIM26. This data further confirmed that USP39 and TRIM26 antagonize each other to control ZEB1 stability and thereby determine the progression of HCC.

### USP39 and TRIM26 function in an antagonistic pattern contribute to the progression of HCC in vivo

**Fig. 8:**
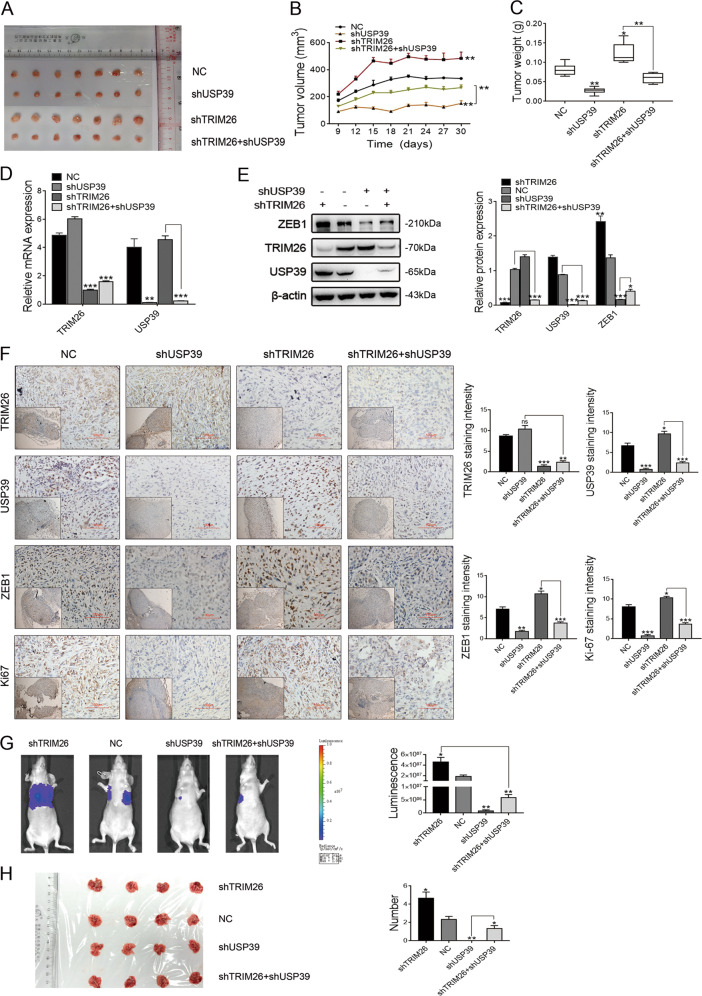
USP39 and TRIM26 function in an antagonistic pattern contributing to the progression of HCC in vivo. **A** The effect of USP39 and TRIM26 in HCC cell proliferation in vivo was determined by xenograft assays. USP39 and TRIM26 knockdown SK-hep-1 cells were respectively injected into flanks of BALB/c nude mice. After 30 days, tumors were isolated and photographed. **B** Tumor volumes were calculated. **C** Tumor weight. **D**, **E** Levels of USP39, TRIM26, and ZEB1 were analyzed by RT-PCR (**D**) and western blotting (**E**). **F** The expression levels of USP39, TRIM26, ZEB1 and Ki67 in tumors of different groups by IHC (original magnification, ×40; inlet, ×10). **G**, **H** Representative images showed the tumors metastasis of different groups by whole-body bioluminescence imaging (**G**) and lung metastases (**H**). The number of nodules in the lung was counted and statistically analyzed. Student’s *t* test: *P < 0.05; **P < 0.01; ***P < 0.001. All the data are representative of at least three independent experiments and presented as the means ± SD.

To assess the effects of USP39 and TRIM26 in the progression of HCC in vivo, USP39 and TRIM26 knockdown SK-hep-1 cells were respectively injected into the flanks of BALB/c nude mice. The downregulation of USP39 expression in HCC cells significantly inhibited tumor growth, by reducing the volume and weight of tumors (Fig. 8A–C). In agreement with this result, tumors derived from shUSP39 cells were much smaller in size than the control group of NC cells. In contrast, the downregulation of TRIM26 expression could remarkably promote the growth of xenograft tumors (Fig. 8A). Furthermore, TRIM26 silencing could significantly reverse the reduction of USP39 knockdown cell proliferation, in tumor size and weight (Fig. 8B, C). Conversely, USP39 knockdown inhibited proliferation in tumor size and weight of SK-hep-1 cells transfected with shTRIM26. The RT-PCR assays and western blot analyses demonstrated that TRIM26 and USP39 levels were downregulated in the shTRIM26 SK-hep-1 cells and shUSP39 SK-hep-1 cells, respectively (Fig. 8D, E). In addition, USP39 knockdown SK-hep-1 cells exhibited decreased ZEB1 and Ki67 protein levels, while TRIM26 downregulation promoted the expression levels of ZEB1 and Ki67 by IHC (Fig. 8F).

To evaluate the effects of USP39 and TRIM26 in the metastatic potential of HCC cells in vivo, SK-hep-1-luc cells with downregulation of USP39 and TRIM26 expression were respectively inoculated into BALB/c nude mice via tail vein injection. Whole-body bioluminescence imaging was employed to investigate the effect of shUSP39 and shTRIM26 on the growth and metastasis of HCC tumors on the 40th day after injection. The results showed that USP39 knockdown led to an obvious reduction in tumor metastasis, while TRIM26 downregulation enhanced the tumor metastasis and reversed the reduction of shUSP39-related cell metastasis and the number of nodules in the lung (Fig. 8G, H). USP39 knockdown also suppressed the shTRIM26-related HCC cell metastasis. Altogether, these results further consolidate a novel mechanism that deubiquitinase USP39 and E3 ligase TRIM26 function in an antagonistic pattern, but not a competitive pattern, and play key roles in controlling ZEB1 stability to determine the HCC progression.

## Discussion

Few therapeutic strategies can improve the OS rate of patients with advanced HCC. Therefore, elucidating the molecular mechanism is vital for the progression of HCC. Deregulation of the deubiquitinase USP39 and the E3 ubiquitin ligase TRIM26 plays an essential role in tumor progression. ZEB1 is a crucial inducer of EMT to promote the metastasis of cancer cells, but whether its stability is regulated by USP39 or TRIM26 in HCC remains enigmatic.

In this study, we demonstrate that USP39 is critically involved in the progression of human HCC. USP39 was overexpressed in clinical HCC samples compared to normal adjacent tissue. The Kaplan–Meier analysis showed that increased expression of USP39 significantly correlated with poor prognosis. Indeed, silencing USP39 expression markedly inhibited the proliferation and metastasis of HCC cells in vitro and in vivo experiments, indicating the potential carcinogenic effect of USP39 in HCC development. Consistent with previous reports, these results further consolidated USP39 as a promoter in tumor progression including HCC. In addition, USP39 downregulation dramatically suppressed the level of ZEB1 as well as EMT progression. Herein, we demonstrate for the first time that the molecular mechanisms of USP39-mediated cell proliferation and metastasis in HCC might be involved in the regulation of the ZEB1-dependent EMT axis. We identified USP39 as a novel and key modulator for ZEB1 in HCC. USP39 is well known as an important modulator of RNA splicing and widely believed to be devoid of deubiquitinating activity. Intriguingly, ZEB1 mRNA showed no major differences in HepG2 transduced with shUSP39, while the level of ZEB1 protein was dramatically suppressed. These results suggest that the mechanism by which USP39 impacts ZEB1 activity is not through transcriptional regulation, but possibly by posttranslational regulation (e.g., ubiquitin degradation). Recently, a report showed that USP39 has a deubiquitination-related function towards CHK2 in lung cancer [19]. Therefore, this controversy related to the deubiquitination function of USP39 needs to be further addressed. In our study, similarly, USP39 can modulate the level of ZEB1 protein via deubiquitination. Also, our results showed that ZEB1 ubiquitination was significantly decreased in SK-hep-1 cells transfected with the USP domains of USP39, which indicated USP39 function as an active DUB for the ZEB1 protein. According to relevant studies [38, 39], USP39 does not have the ability to self-deubiquitinate, but it can form a stable complex with USP4 to remove the inhibition of its catalytic activity and then activate the deubiquitinase activity of USP4. Therefore, the interference factors affecting the deubiquitination of USP39 need to be investigated in the near future. Collectively, we believe that USP39 reduces the degradation of ZEB1 protein through direct deubiquitination, which promotes EMT progression and the development of HCC.

Ubiquitination and deubiquitination are two contrasting posttranslational processes that involve in the conjugation and removing of ubiquitin from targeted proteins, respectively. TRIM26 has an E3 ubiquitin ligase activity, which has been found to significantly inhibit cell proliferation and migration in HCC. It is interesting to speculate whether E3 ligase TRIM26 with ubiquitin function has an interaction with deubiquitinase USP39 to control the stability of ZEB1 protein. Co-immunoprecipitation assays in our study showed that USP39 directly interacts with TRIM26, and immunofluorescence staining assays revealed that USP39 and TRIM26 co-localized in the nucleus of cells (SK-hep-1 and HepG2). These observations suggested that deubiquitinase USP39 could interact with E3 ligase TRIM26 by which the progression of HCC may be affected. TRIM26 is an E3 ligase enzyme which mediates ubiquitination and degradation of many proteins, such as IRF3 [40, 41]. In addition, a microarray expression profiling in our study showed that the expression of TRIM26 was lower in HCC tissues compared with normal adjacent tissue. HCC patients with a high level of TRIM26 mRNA had a better OS than those with low TRIM26 expression. Our studies in vitro showed that a decreased TRIM26 expression could elevate the expression level of ZEB1 and additionally promote the proliferation and migration of HCC cells. Previous studies held the same view that TRIM26 could represent a novel therapeutic target for HCC [24]. Meanwhile, we identified ZEB1 as the potential ubiquitination-associated protein of TRIM26 interaction. Therefore, we found that USP39 and TRIM26 not only directly interact with each other, but also have a common target (ZEB1). We hypothesized that USP39 antagonizes TRIM26 in targeting ZEB1 for ubiquitin-dependent proteasomal degradation, to regulate the proliferation and migration of HCC cells. This hypothesis was further corroborated by the fact that USP39 could reverse the effect of TRIM26 on ZEB1 expression and the progression of HCC. Similarly, TRIM26 also could reverse the effect of USP39 on ZEB1 expression and the proliferation and migration of HCC cells. Altogether, these results further consolidate a novel mechanism that deubiquitinase USP39 and E3 ligase TRIM26 function in an antagonistic pattern and play key roles in controlling ZEB1 stability to determine the HCC progression. These results are similar to those of Nicklas et al. [42] where c-Myc expression is controlled by E3 ligase TRIM32 and deubiquitinase USP7, and the net balance between protein ubiquitination and deubiquitination acts as a key determinant for cancer development. Of course, Luo et al. also reports that E3 ligase TRIM32 and the deubiquitinase USP11 balance ARID1A stability to regulate squamous cell carcinoma cell proliferation and metastasis [43].

In summary, we report a modification via an elaborate balance between ubiquitination and deubiquitination as a novel mechanism for regulating the progression of HCC. The regulation of this molecular process is deubiquitinase USP39 and E3 ligase TRIM26 balancing the level of ZEB1 ubiquitination and thereby determine HCC cell proliferation and migration. This novel mechanism may provide new approaches to target treatment for inhibiting HCC development by restoring TRIM26 or suppressing USP39 expression in HCC cases with high ZEB1 protein levels.

## Supplementary information

Supplementary Table S1

Supplementary Fig. S1

Supplementary Fig. S2
