# Supplementary material for: Deubiquitinase USP39 and E3 ligase TRIM26 balance the level of ZEB1 ubiquitination and thereby determine the progression of hepatocellular carcinoma
Source: Cell Death Differ. 2021 Mar 1;28(8):2315–32. doi: 10.1038/s41418-021-00754-7 (PMC8329202; doi:10.1038/s41418-021-00754-7)
Supplement: Supplementary file 1 — Supplementary Table S1 [file 41418_2021_754_MOESM1_ESM.doc]

Tables S1

**Table S1** Primers for qRT-PCR

| Genes | Primers | Sequences (5’-3’) |
| --- | --- | --- |
| *trim26* | F | TGCACTACTACTGTGAGGACG |
|  | R | TCCTTAGGGTACTCAGGTGGT |
| *usp39* | F | TTGGAAGAGGCGAGATAA |
|  | R | AGGAGCATCAATCATCATC |
| *zeb1* | F | CAGCTTGATACCTGTGAATGGG |
|  | R | TATCTGTGGTCGTGTGGGACT |
| *gapdh* | F | TGCACCACCAACTGCTTAGC |
|  | R | GGCATGGACTGTGGTCATGAG |
