# Supplementary figures and images for: Deubiquitinase USP39 and E3 ligase TRIM26 balance the level of ZEB1 ubiquitination and thereby determine the progression of hepatocellular carcinoma
Source: Cell Death Differ. 2021 Mar 1;28(8):2315–32. doi: 10.1038/s41418-021-00754-7 (PMC8329202; doi:10.1038/s41418-021-00754-7)

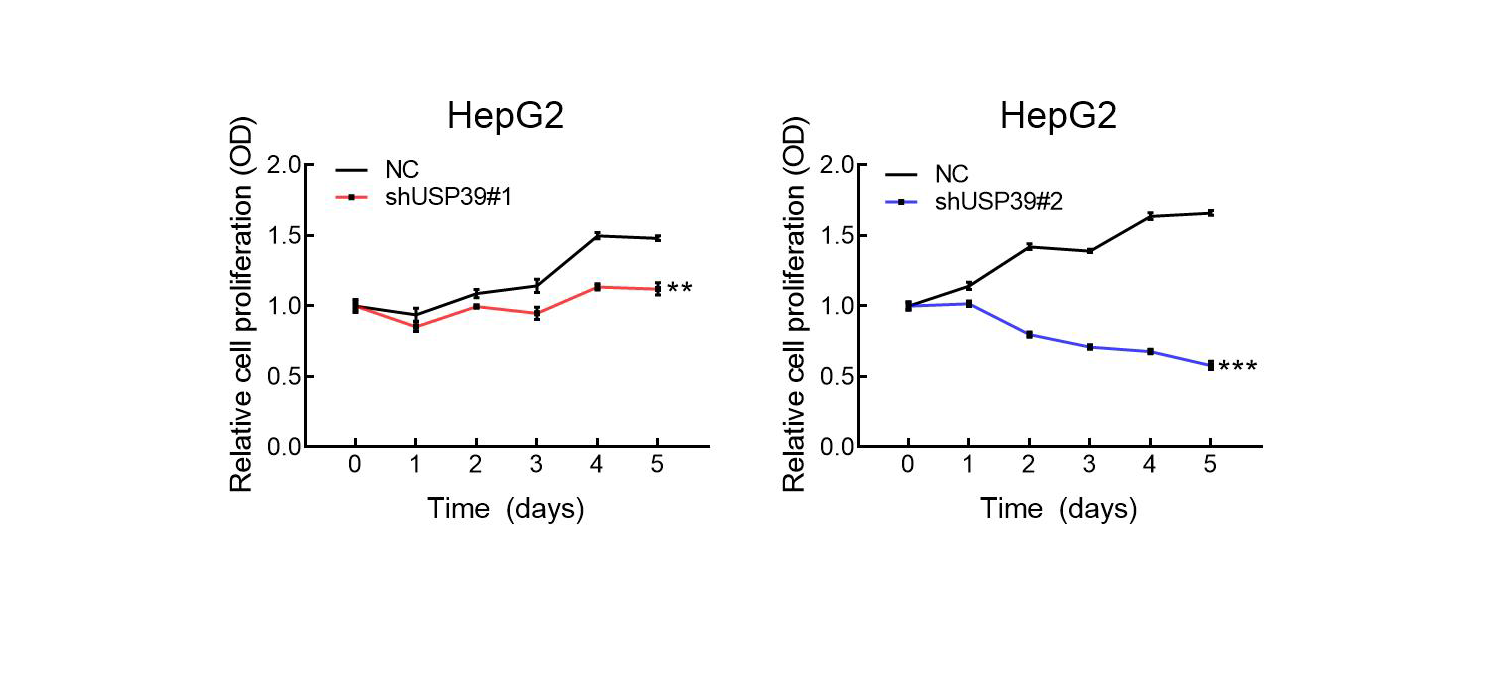

Supplement: Supplementary file 2 — Supplementary Fig. S1 [file 41418_2021_754_MOESM2_ESM.tif]

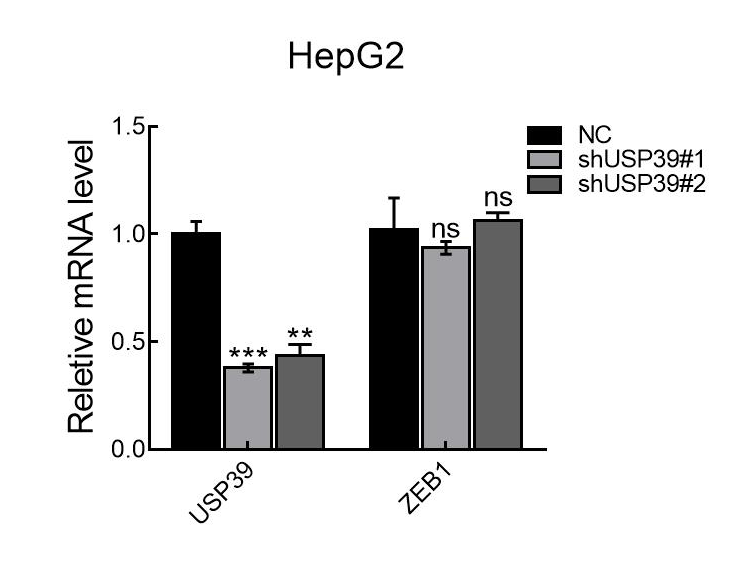

Supplement: Supplementary file 3 — Supplementary Fig. S2 [file 41418_2021_754_MOESM3_ESM.tif]
